# Quantitative Analysis of a Weak Correlation between Complicated Data on the Basis of Principal Component Analysis

**DOI:** 10.1155/2021/8874827

**Published:** 2021-01-20

**Authors:** Tao Pang, Haitao Zhang, Liliang Wen, Jun Tang, Bing Zhou, Qianxu Yang, Yong Li, Jiajun Wang, Aiming Chen, Zhongda Zeng

**Affiliations:** ^1^Yunnan Academy of Tobacco Agriculture Science, Yuxi, Yunnan 653100, China; ^2^China Tobacco Yunnan Industrial Co., Ltd., Kunming, Yunnan 650202, China; ^3^Dalian ChemDataSolution Information Technology Co. Ltd., Dalian 116023, China; ^4^College of Environmental and Chemical Engineering, Dalian University, Dalian 116622, China

## Abstract

The mining of weak correlation information between two data matrices with high complexity is a very challenging task. A new method named principal component analysis-based multiconfidence ellipse analysis (PCA/MCEA) was proposed in this study, which first applied a confidence ellipse to describe the difference and correlation of such information among different categories of objects/samples on the basis of PCA operation of a single targeted data. This helps to find the number of objects contained in the overlapping and nonoverlapping areas of ellipses obtained from PCA runs. Then, a quantitative evaluation index of correlation between data matrices was defined by comparing the PCA results of more than one data matrix. The similarity and difference between data matrices was further quantified through comprehensively analyzing the outcomes. Complicated data of tobacco agriculture were used as an example to illustrate the strategy of the proposed method, which includes rich features of climate, altitude, and chemical compositions of tobacco leaves. The number of objects of these data reached 171,516 with 14, 4, and 5 descriptors of climate, altitude, and chemicals, respectively. On the basis of the new method, the complex but weak relationship between these independent and dependent variables were interestingly studied. Three widely used but conventional methods were applied for comparison in this work. The results showed the power of the new method to discover the weak correlation between complicated data.

## 1. Introduction

Information extraction and mining of data with high complexity has increasing interests in both academic and industrial sectors. With rapid development and use of smartphones and sensing equipments and advanced scientific instruments such as chromatography, mass spectrometry, spectroscopy analysis and their coupled techniques, theoretical calculations, and simulation, of course, the difficulty of generation of data has been largely overcome to date [1–5]. Thus, it has strong priority to further develop powerful algorithms for knowledge discovery according to the characteristics of different types of data themselves. The conventional strategies are not always flexible to mine rich information hidden in datasets. Using a typical data of the tobacco planting process as an example, the quality of tobacco leaves, such as the chemical compositions, and physical properties, such as consuming experience, are inevitably affected by ecological conditions, soil, planting, and production processes of tobacco. Of course, the genes and mutations of tobacco are also potential factors to the results [6–8]. However, huge challenges may be encountered while attempting to discover the influence of independent variables/factors on dependent indices/indicators. The reason is that there are many different kinds of data types or sources, and the unknown relationship between various variables is quite complicated and not easy to be discovered. This leads to the difficulty for reasonable interpretation on the basis of the conventional methods for univariate or multivariate analysis [9, 10].

Traditionally, the process for quantitative modeling is applied as follows. First, univariate statistical analysis of each feature in a single data is performed, and hypothesis testing between different types of samples is used to obtain statistical results of a single feature. This helps to find and further remove the outliers from both the dimensions of objects and variables [11]. Next, data exploratory analysis methods such as principal component analysis (PCA) are used to mine the correlation between data matrices, but one of the main problems is the difficulty to provide quantitative results for evaluation. This mostly generates a model with low generalization ability and poor conclusions, especially to the matrices with weak correlation [12–14]. Furthermore, the methods for classification or regression analysis can be applied to build a multivariate model for qualitative or quantitative analysis, if required. This kind of analysis can help to correlate the relationship between independent and dependent variables [15–18]. Of course, other rich types of methods, such as Boolean association rules, decision trees, recommendation algorithms, and deep learning, can also be utilized to attain the goals [19–22]. In addition, canonical correlation analysis seeks to find the correlation between comprehensive data pairs and reflect the overall correlation between them. That is, it discovers the correlation between data matrices as a whole and extracts the representative information of data by using canonical variates. But, it is still incapable to correlate the data with low internal connection [23, 24].

Many researchers reported the studies to correlate the relationship between multiple datasets. PCA should be one of them with the highest concerns used in different fields, which has also been extensively developed in theory and applications, including sparse PCA generated by an elastic network (LASSO), probabilistic method on the basis of an associated likelihood function, and the robust PCA for processing of data with outliers [25–28]. Since it helps for financial decision makers to deal with credit classification problems, the credit classification model has been largely applied in recent years. Tang et al. were inspired by the nonlinearity of dendrites in biological neural models and proposed a pruned neural network and applied to solve classification problems. The results showed that it was superior to other classical algorithms in terms of accuracy and computational efficiency [29]. Using the processing of heavy metal adsorption as an example, Bingöl et al. proposed a nonlinear autoregressive network with exogenous inputs (NARX) and compared with multiple regression analysis (MLR). It was found that the prediction ability of the NARX method was superior to the MLR method using dummy variables, which can successfully achieve the evaluation of the adsorption process on the experimental data [30]. Canonical correlation analysis (CCA) can be used to study the correlation between two datasets, which is a classical statistical tool to correlate multivariate data. Jendoubi et al. proposed a CCA probabilistic model in the form of a two-layer latent variable model and used for integrated analysis of gene expression data, lipid concentration, and methylation level omics datasets. It provided a new strategy to unify the spheroidization process, multiple regression, and the corresponding probability model [31]. In addition, deep learning has been widely applied in many areas for big data analysis. Litjens et al. introduced its applications in image classification, target detection, segmentation, and registration for the analysis of the nerve, retina, lungs, digital pathology, breast, heart, abdomen, and so on [32].

In this work, a new method called PCA/MCEA (PCA-based multiconfidence ellipse analysis) was developed for correlation analysis, which was based on PCA to discover weak information between datasets. It first utilized PCA for dimensionality reduction after data quality improvement. The confidence ellipse analysis then quantitatively analyzed the similarity and difference among different categories of samples. Next, a quantitative index was defined to introduce the correlation of samples in terms of the sample/objects distribution in the individual ellipse of each sample class, as well as their results in the overlapping zone of the ellipses. In the end, the correlation between independent variables and dependent variables was attained after comprehensively analyzing the performance of these findings between different samples. The findings showed that the PCA/MCEA method can be used as an effective tool to mine out the correlation information between multidimensional data with high complexity. Example data of tobacco agriculture were applied to deliver the strategy, which included planting climate and altitude, and the corresponding concentrations of chemical compositions. The results of visualization analysis further explained the findings and show rich characteristics and relationships of planting production of tobacco collected in different locations/regions. Three traditional methods were used for comparison. The strategy and procedure of the PCA/MCEA method can be extensively used for analysis of other types of datasets.

## 2. Theory

### 2.1. Principal Component Analysis

PCA has been widely employed for data processing, which has the power to reduce data size by projecting the raw data to low-dimensional space containing most of the original variance and ignoring part of the features with small variance [12, 27]. The PCA method can be used for both data compression and extraction and removal of interference factors.

Singular value decomposition (SVD) is a strategy to realize PCA analysis to obtain the orthogonal principal components (PCs) with the results shown in the following equation [27]:

(1) $$\begin{array}{c} A=U\Sigma V^{T}, \end{array}$$

where *A* denotes the raw data with a size of *m* × *n* for decomposition and the three matrices *U*, Σ, and *V*^*T*^ denote scores, singular values, and loadings with sizes of *m* × *m*, *m* × *n*, and *n* × *n*, respectively.

### 2.2. Multidimensional Confidence Ellipse

As described above, the three matrices *U*, *V*^*T*^, and Σ obtained after PCA operation, respectively, represent the left and right singular vector and singular values of original data *A*. Through analyzing these matrices, the correlation and difference between objects/samples in *A* can be discovered. The significant difference amongst these objects or features of *A* can be achieved with the help of confidence interval analysis [33]. Using two-dimensional data as an example, the score plot with the first two or three PCs can be constructed based on matrix *U*, and furthermore, the confidence ellipse, for example, 95% ellipse on the plot, can be obtained to show the distribution of samples inside or out of the ellipses or included inside of the overlapping zone of two ellipses. Then, multiple confidence ellipses of different types of samples can be applied to deeply mine out the correlation of distribution and further find the characteristics of samples and the correlation and difference among these samples. Typically, the confidence ellipse analysis utilizes the smallest ellipse that covers 95% of data points (objects/samples) in the score plot to a certain class of samples. The ellipse has two important parameters, that is, area of the ellipse and inclination of the principal axis relative to the *x*-axis or *y*-axis in the plot representing the direction of change of the ellipse. The ellipse can be calculated by assuming an approximate Gaussian distribution of the objects coordinating around its average value.

### 2.3. PCA/MCEA Method

**Figure 1:**
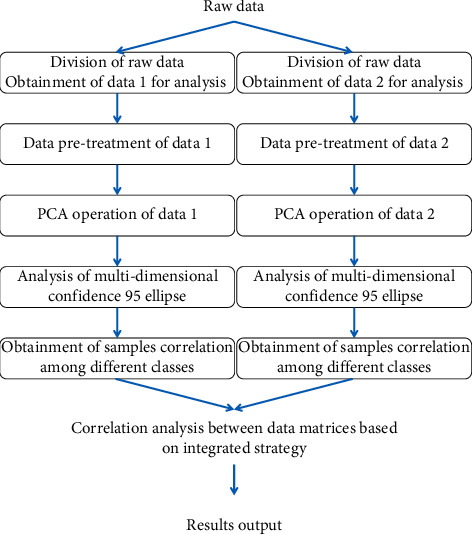
The flowchart of the PCA/MCEA method for data analysis.

In this paper, a quantitative method to evaluate the correlation of two matrices was proposed on the basis of PCA operation, and thus, it was originally named as multidimensional confidence ellipse analysis (PCA/MCEA). The flowchart of the PCA/MCEA method is shown in Figure 1.

First, the original data are divided into multiple matrices according to the actual situation of data information, which is included in the introduction section of matrix. This step is applied to obtain the multiple data for processing and correlation assessment. After this, the PCA method is independently performed to reduce the dimensionality of each data. After this, determination of class of samples can be attained by using the priori knowledge. Next, the multidimensional confidence ellipse analysis can be used to analyze the independent variables between samples of each category and similarity of the dependent variables. The potential correlation between independent variables and dependent variables, namely, integrated analysis of different matrices to be analyzed, can be achieved on the basis of these results.

#### 2.3.1. Division of Raw Data with More Than One Data Matrix

In terms of the source and type of different indexes of the original data, it is divided into several matrices for correlation analysis. Using data with size of *n* × *m* as an example, the original data can be expressed as follows:

(2) $$\begin{array}{c} \Psi =\left\{X,Y,D\right\}. \end{array}$$

The three matrices *X*={*x*_1_,…, *x*_*m*_1__}, *Y*={*y*_1_,…, *y*_*m*_2__}, and *D*={*d*_1_, *d*_2_,…, *d*_*m*_3__}, respectively, represent data with independent variables, dependent variables, and sample description information. The sizes of the three matrices are *m*_1_, *m*_2_, and *m*_3_. The independent variables and dependent variables of data *X* and *Y* can be further subdivided, as shown in equations (3) and (4).

(3) $$\begin{array}{c} X=\left\{X_{1},X_{2},\ldots ,X_{M_{1}}\right\}, \end{array}$$

(4) $$\begin{array}{c} Y=\left\{Y_{1},Y_{2},\ldots ,Y_{M_{2}}\right\}, \end{array}$$

where *X*_1_ ∪ *X*_2_ ∪ ⋯∪*X*_*M*_1__=*X*, *X*_*i*_∩*X*_*j*_=*ϕ*(*i*, *j*=1,2,…, *M*_1_, *i* ≠ *j*), *Y*_1_ ∪ *Y*_2_ ∪ ⋯∪*Y*_*M*_2__=*Y*, and *Y*_*i*_∩*Y*_*j*_=*ϕ*(*i*, *j*=1,2,…, *M*_2_, *i* ≠ *j*). After these processing steps, two matrices of independent variables and dependent variables can be mathematically obtained for analysis.

#### 2.3.2. Dimensional Reduction Analysis by Using PCA

As introduced above, the PCA operation can be used for analysis of two datasets with independent variables and dependent variables, and the PCs with the largest variance can be extracted with removal of interference information.

Supposing the data to be analyzed as *Z*=*X*+*Y*, the submatrix *Z*_*i*_ ∈ *Z*(*i*=1,2,…, *M*_1_, *M*_1_+1,…, *M*_1_+*M*_2_) is decomposed and the latent variables with the largest variance are selected for subsequent analysis.

(5) $$\begin{array}{c} k=\underset{Z_{i}\in Z}{\max}\left({\text{MinPriCol}}_{\sigma }\left({\left\|Z_{i}\right\|}_{\text{pca}}\right)\right), \end{array}$$

where ‖*Z*_*i*_‖_pca_ represents results of *Z*_*i*_ by using PCA and MinPriCol_*σ*_(‖*Z*_*i*_‖_pca_) represents the minimum number of PCs, in which cumulative variance contribution exceeds to a predefined threshold *σ*. That is to say, the cumulative variance contribution of the first *k* PCs of ∀‖*Z*_*i*_‖_pca_(*i*=1,2,…, *M*_1_, *M*_1_+1,…, *M*_1_+*M*_2_) is greater than the threshold *σ*, and ∃‖*Z*_*i*_‖_pca_, the cumulative variance contribution of the first *k* − 1 PCs, is less than the threshold *σ*.

Finally, the first *k* latent variable after PCA analysis is extracted to construct a new dataset of latent variable *T*={*T*_*i*_}(*i*=1,2,…, *M*_1_, *M*_1_+1,…, *M*_1_+*M*_2_), in which the matrix *T*_*i*_ includes the first *k* columns of ‖*Z*_*i*_‖_pca_.

#### 2.3.3. PCA Analysis to Each Class of Samples

According to the sample description information *d* ∈ *D*, *d* classes of samples can be divided for analysis. Similarly, each data matrix *T*_*i*_ ∈ *T* can be divided into *D*={*D*_*j*_}(*j*=1,2,…, |*d*|) numbers of classification. Then, a total of (*M*_1_+*M*_2_) × |*d*| data matrices were analyzed to a data matrix with *M*_1_+*M*_2_ independent and dependent variables and recorded as *P*={*P*_*i*_}. The set *P*_*i*_={*P*_*ij*_} (*j*=1,2,…, |*d*|) is the |*d*| classification result.

#### 2.3.4. Analysis of the Multidimensional Confidence 95 Ellipse

**Figure 2:**
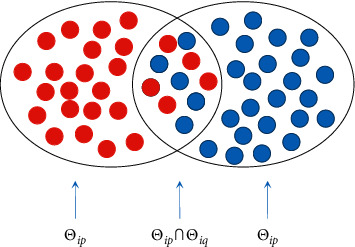
Schematic diagram of confidence ellipse analysis included in the proposed method, in which a two-dimensional confidence ellipse example is given to deliver the strategy. Here, the two ellipses represent two classes of samples, and the data points with two different colors denote samples corresponding to different classes. The overlapping and nonoverlapping areas of ellipses include the common and noncommon characteristics of the two types of samples.

Based on the results of PCA operation, the multidimensional confidence ellipse of each data matrix *P*_*ij*_(*i*=1,2,…, *M*_1_, *M*_1_+1,…, *M*_1_+*M*_2_, *j*=1,2,…, |*d*|) will generate (*M*_1_+*M*_2_) × |*d*| number of multidimensional ellipses. It is denoted as Θ_*i*_={Θ_*ij*_}, in which Θ_*i*_={Θ_*ij*_}(*j*=1,2,…, |*d*|) represents the multidimensional ellipse corresponding to the |*d*| numbers of sample classification, as shown in Figure 2.

For the |*d*| ellipses in the multidimensional confidence ellipse {Θ_*ij*_}(*i*=1,2,…, *M*_1_, *M*_1_+1,…, *M*_1_+*M*_2_, *j*=1,2,…, |*d*|) corresponding to data *P*_*i*_ of classification *Z*_*i*_, it can be divided into two spaces, that is, inside and out of the space. Through statistical analysis of sample classification *D*_*p*_, *D*_*q*_(*p*, *q*=1,2,…, |*d*|, and *p* ≠ *q*), the number of different types of samples existing in the confidence ellipse Θ_*ip*_ and Θ_*iq*_ respectively, describe the classification information of *Z*_*i*_, sample classification *D*_*p*_ and *D*_*q*_, and the degree of sample internal aggregation corresponding to the quantification of similarity and difference evaluation of samples of *D*_*p*_ and *D*_*q*_.

The quantitative index for evaluation is provided as follows:

(6) $$\begin{array}{c} S_{i,p,q}=\frac{{\left\|\Theta_{ip}\cap \Theta_{iq}\right\|}_{p}}{\left\|D_{ip}\right\|}, \end{array}$$

where ‖*D*_*p*_‖ indicates *D*_*p*_ number of samples in sample classification and ‖Θ_*ip*_∩Θ_*iq*_‖_*p*_ indicates the number of samples simultaneously existing in sample classification *D*_*p*_ and *D*_*q*_, which corresponds to the overlapping area in the multidimensional confidence ellipse. *S*_*i*,*p*,*q*_ denotes the similarity between sample classes of *D*_*p*_ and *D*_*q*_ in the case of classification *Z*_*i*_. In particular, it indicates the degree of aggregation of the sample classification *D*_*p*_ in the case of index classification *Z*_*i*_, if *p*=*q*.

By using the multidimensional confidence ellipse analysis method introduced above, the similarity and difference among multiple classes of samples can be quantitatively evaluated, and this helps to discover the characteristics of sample classification, such as *Z*_*i*_ here.

#### 2.3.5. Integrated Correlation Analysis among More Than One Data Matrix

The PCA/MCEA method quantitatively analyzes the results of each classification of samples *Z*_*i*_ (*i*=1,2,…, *M*_1_, *M*_1_+1,…, *M*_1_+*M*_2_), degree of aggregation of different types of samples, and similar characteristics between classes. This helps to determine the association between multiple data matrices. By the confidence ellipse analysis of different classes of samples and then the correlation coefficient between the number of samples included in the ellipse of each class, the correlation between data can be discovered by analyzing the number of different groups of samples. It is especially helpful to mine out weak correlation, for example, while the classification or regression relationship is not significant.

The proposed method is unlike the traditional strategies for direct analysis of correlation of independent variables (*X*) and dependent variables (*Y*), which seeks to find the simple relationship. This is probably unsuitable for data matrices with a weak relationship. The PCA/MCEA method completely avoids the difficulties and challenges encountered in constructing multivariate classification or regression models, but constructs a confidence ellipse to analyze relationship after PCA analysis. The samples included in the overlapping and nonoverlapping samples of the ellipse effectively denote the correlation of similarity and difference between different classes of samples. After this, the correlation between more than one matrix can be found through individually analyzing the contribution of different independent variables on the influence of dependent variables.

### 2.4. Conventional Methods

In this study, stepwise regression analysis, PLS regression analysis, and SVR regression analysis methods were used for comparison of the proposed method.

#### 2.4.1. Stepwise Regression Analysis

In the process of stepwise regression analysis, an independent variables *X*_*i*_ is introduced in each run, and the regression coefficients must be tested by the *F* test. It is recorded as *F*_1_^(1)^,…, *F*_*p*_^(1)^, respectively, and it is assumed that *F*_*i*_1__^(1)^=max{*F*_1_^(1)^,…, *F*_*p*_^(1)^}. The independent variable is introduced to the regression model, if *F*_*i*_1__^(1)^ is greater than the critical value *F*^(1)^, which corresponds to the given significance level *α*. Otherwise, it will be excluded to the model [34].

The stepwise regression method realizes the screening of “optimal” independent variables by gradually introducing variables and further calculates the square sum of partial regression analysis. It avoids the multicollinearity problem that occurs while using full independent variable analysis.

#### 2.4.2. Partial Least Squares Regression Analysis

In PLS regression analysis, the independent and dependent variables are projected into a new space to generate a linear regression relationship in a new space [35]. The PLS regression analysis method avoids structural uncertainty and nonnormal distribution problems by extracting the maximum information reflecting data variability.

#### 2.4.3. Support Vector Regression Analysis

The support vector regression (SVR) analysis uses the optimal model shown in formula (7), which helps to find the hyperplane with the “shortest distance” from the farthest sample to the hyperplane [36].

(7) $$\begin{array}{c} \left\{\begin{array}{c} \min\frac{1}{2}{\left\|w\right\|}^{2},\\ \text{s.t}.\;\left|yi\left(wx_{i}+b\right)\leq \varepsilon \right|\forall i. \end{array}\right. \end{array}$$

The SVR regression analysis is the first on the basis of structured risk minimization, which introduces an *ε*-insensitive loss function. Especially, it has strong generalization ability to reduce the requirement for balanced data sampling of samples.

## 3. Data Introduction and Analysis

In this article, an example data of tobacco agriculture was employed to deliver the strategy proposed in this work. The purpose is to study the impact of an ecological environment, including climate and altitude, on the quality of tobacco leaves, which was originally collected from Yunnan Province, one of the largest districts for tobacco planting in China. The detailed information of the dataset is given in Table 1. In this table, the climate and altitude indices and chemical compositions for quality evaluation of tobacco are introduced. It totally includes 14, 4, and 5 indices of the three independent and dependent variables. The total number of samples reaches 171,516.

Before processing, the data quality was improved by using the following steps, including filling of missing values, removal of outliers, and data normalization:

  Deletion of missing values: the samples with missing values of climate, altitude, and/or chemicals of tobaccos were eliminated before the next step.
  Removal of outliers: the strategy of box-plot was applied to remove the samples of outliers.
  Data normalization: the Z-score method was used for data normalization. That is, the mean (*μ*) and variance (*σ*) of the each variable were calculated and standardized according to the following formula: *x*′=(*x* − *μ*)/*σ*.

After these steps, a total of 168,643 sample data are finally generated for analysis.

## 4. Results and Discussion

### 4.1. Workflow of the PCA/MCEA Method

To use the PCA/MCEA method for analysis, a total of 168,643 samples were preprocessed as introduced above, and then, they was divided into 35 classes of samples on the basis of the geographic locations attributes of the 35 “county and city/district” of samples. In terms of the PCA/MCEA method introduced in Figure 1, the preprocessed data were processed for analysis.

The specific parameters and processing factors of the PCA/MCEA method are described as follows:

1. The original data was divided and reduced into independent variables including climate (*X*_1_), altitude (*X*_2_), and dependent variables, including 5 indices of tobacco (*Y*_1_)
2. For PCA analysis, the cumulative variance threshold was defined as *σ*=0.8 for the three data matrices *X*_1_, *X*_2_, and *Y*_1_ during dimensionality reduction
3. In terms of the sample description information, including “county and city/district” index, the 168,643 real samples were divided into 35 classes for the subsequent confidence ellipse analysis
4. The final integrated analysis of correlation was attained on the basis of these results and findings, in which the influence of independent variables to dependent variables was introduced.

**Figure 3:**
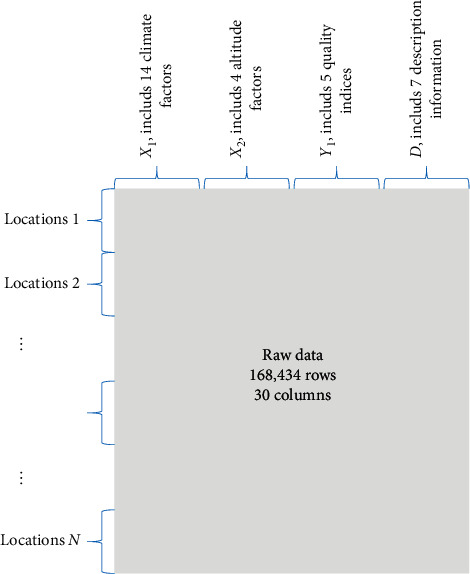
The structure of original data applied as an example to introduce the strategy of the PCA/MCEA method.

**Figure 4:**
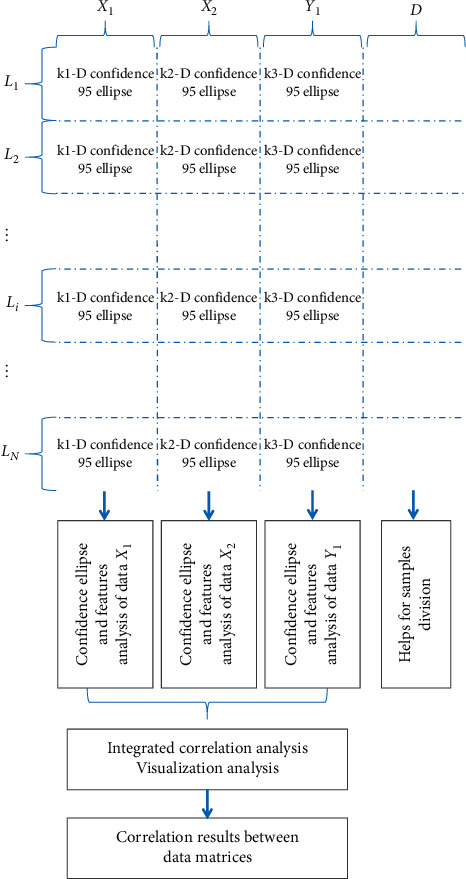
The specific scheme of the proposed method with the help of multidimensional confidence ellipse analysis.

The data structure of experimental data is shown in Figure 3, and the process for PCA/MCEA analysis is shown in Figure 4.

### 4.2. Results of the PCA/MCEA Method

**Figure 5:**
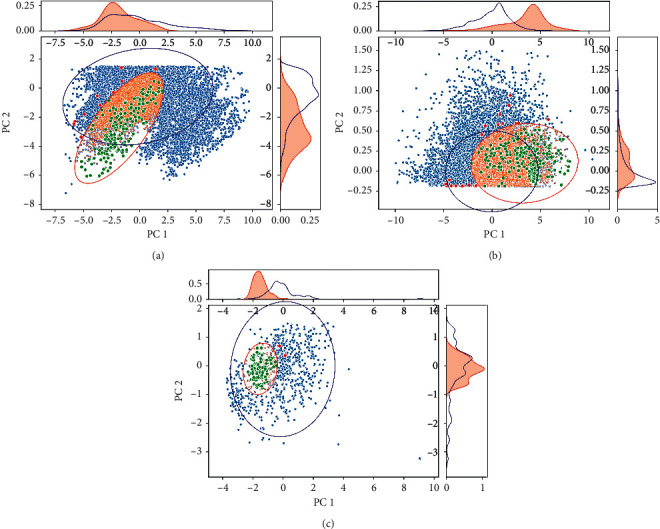
Using the samples of GCQ as an example, the results of 2-dimensional confidence ellipse analysis of climate, altitude, and chemical compositions of tobacco leaves were generated. (a), (b), (c) The results of 2-dimensional confidence ellipse analysis by using the data of climate, altitude, and chemical compositions of tobacco leaves. In the three plots, the red ellipse is constructed by using the samples of GCQ, and the blue one is constructed by using all samples of other locations. Each green data in the red ellipse denotes a sample of GCQ, and the red point in the area denotes the samples of other areas. Correspondingly, the samples of GCQ excluding in this ellipse are shown in red color, and samples of other areas are shown in blue color.

The three datasets of climate (*X*_1_), altitude (*X*_2_), and chemical compositions of tobacco leaves (*Y*_1_) were analyzed by the PCA operation. Based on the cumulative variance threshold, the new variables with the largest variance were selected for analysis. As shown in Figure 5, the results were obtained by using the PCA/MCEA method, which is obtained by comprehensively analyzing each data with the help of PCA and confidence ellipse. The results of PCA fully show the distribution of samples obtained from different geographic locations. In Figures 5(a)–5(c), each plot corresponds to three different parts, in which the main results in the middle of the figure are obtained from PCA analysis, and the points of two different colors correspond to the samples of targeted category and all the remainings outside of the target class. The common information of each class was explained by the overlapping zone of the 2-dimensional confidence ellipses, as described above. The results shown in the top and right subgraph are on the basis of the results of each class of objects, which were extracted from different categories/geographic regions. The distribution density of the samples is constructed from the first PC and the second PC, respectively. Obviously, the results of different categories of samples can be well identified and distinguished from the distribution of the two curves of density. If the two categories of samples were well distinguished, the overlapping area will be smaller in the curve of density distribution, and *vice versa*. If the samples of the same category of samples were more concentrated, the curve will be sharper with a small value of standard deviation (SD). That is, SD of the curve is smaller, and *vice versa*. The results of Figures 5(a)–5(c) correspond to the analytical results of climate, altitude, and chemical compositions, respectively.

A specific class of sample, namely, GuChengQu (the ancient district of a city and abbreviated as GCQ), was applied as an example to illustrate the process for quantitative analysis of data with weak correlation by using the PCA/MCEA method. As mentioned above, Figures 5(a)–5(c) are the samples of GCQ and all other counties and cities except GCQ and the 2-dimensional confidence ellipse distribution after PCA operation. The results in Figure 5 show that the samples of GCQ have certain unique characteristics in terms of climate, altitude, and chemical compositions of tobacco leaves.

**Figure 6:**
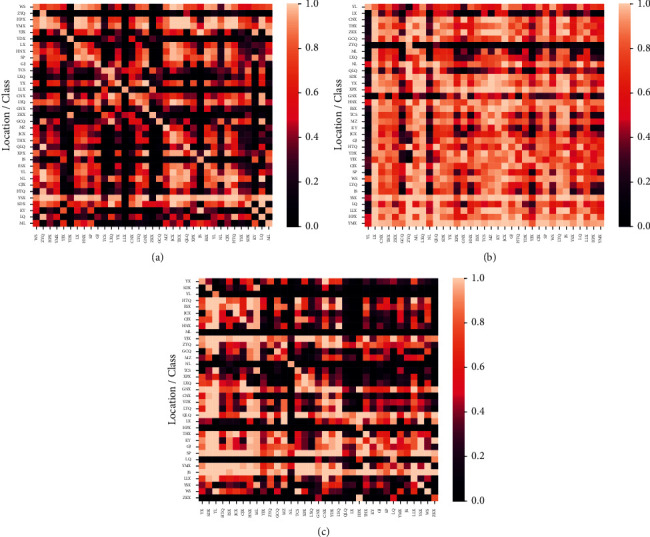
The correlation results of data of climate, altitude, and chemical compositions correspond to the 35 locations. (a), (b), (c) The results of climate, altitude, and chemical compositions of the 35 locations. Each grid denotes the correction results corresponding to the samples existing in the class of the i^th^ row and j^th^ column. The row and column can be obtained from the corresponding geographic locations.

On the basis of the formula given in (2)–(5), the results of 2-dimensional confidence ellipse analysis were obtained to the samples of 35 locations, respectively, corresponding to the data matrices of climate, altitude, and chemical compositions, and the confidence ellipses analysis were, respectively, attained. The number of samples distributing inside and out of these ellipses were further analyzed, and the results are shown in Figure 6. In Figures 6(a)–6(c), the values in diagonal of the three heat maps are larger, of course, which indicates that the samples of climate, altitude, and chemical compositions have higher autocorrelation in the 35 locations/classes. However, the values may not reach 1, since the samples of a class are not completely included inside of the class. From the overall view of the three maps, the overall evaluation of samples located in the 35 zones should be quite similar, but there may exist a large difference in certain areas of samples in climate and chemical composition. In Figure 6(a), the data of GCQ have a higher correlation with Linxiang District (LXQ), Luquan County (LQX), Zhanyi District (ZYD), Hongta District (HTQ), and Ninglang County (NLX), with the correlation coefficient of the index defined in equation (6) reaching 0.96, 0.95, 0.91, 0.89, and 0.85, respectively. These values represent the proportion of all the GCQ samples in the overlapping area of the confidence ellipse analysis between the GCQ and the other areas in the PCA results of climate data. That is to say, it indirectly represents the common characteristics of the samples of GCQ and samples of other regions. The higher the proportion of shared samples, the more the information contained between samples of different locations. Similarly, the results of GCQ has the highest correlation with Zhanyi District (ZYD), Qilin District (QLD), Jiangchuan County (JCX), Luxi County (LXX), and Linxiang District (LXD) in Figure 6(b), with the correlation coefficient of the index defined in equation (6) reaching 1.0, 1.0, 0.99, 0.99, and 0.98, respectively. The results express the correlation of samples in different geographic areas by using the data of altitude. In Figure 6(c), the samples the GCQ have a higher correlation with Yunlong County (YLX), Mile County (MLX), Huaning County (HNX), Shidian County (SDX), and Jiangchuan County (JCX), with the correlation coefficient of the index defined in equation (6) reaching 1.0, 0.96, 0.88, 0.87, and 0.76, respectively. On the other hand, it is also possible to analyze the uncorrelation between the GCQ and other regions, as well as the analysis of other geographic regions, as shown in Figures 6(a)–6(c).

Furthermore, we conduct an integrated analysis of the correlation between the three data on the basis of the correlation coefficients defined in Figures 6(a)–6(c) in terms of the strategy of the PCA/MCEA method. The correlation between each two data of a specific location, for example, the data of GCQ, can be calculated and then used to analyze the quantitative impact of climate and altitude on the chemical compositions of tobacco leaves. To the data pair of climate and chemicals, the minimum, average, and maximum correlation coefficients are −0.2796, 0.0320, and 0.3334, respectively. To the data pair of altitude and chemicals, the three correlation coefficients are −0.2610, 0.0759 and 0.3593, respectively. To the data pair of climate and altitude, the values of these three correlation coefficients are −0.1718, 0.1612, and 0.4717, respectively. Using the results of GCQ as an example, the abovementioned three correlation coefficients are 0.33073, 0.07855, and 0.26514. The results of GCQ show that the climate has a more significant effect on the change quality of tobacco leaves, compared with the altitude factor. Of course, it is particularly important to notice that there is a potentially nonlinear relationship between climate and altitude on the quality of tobacco leaves. The characteristics of different regions are quite different. In addition, there are still too many other kind of factors with possible influence to the quality of tobacco leaves. The extent of a specific factor may be not completely the same to tobacco quality. The advantage of the PCA/MCEA method is that it attempts to quantitatively analyze the relationship between the influencing factors of the two groups of dependent variables and the independent variables from a perspective of a single group of sample. This has certain advantages and application prospects in contrast to the conventional methods.

**Figure 7:**
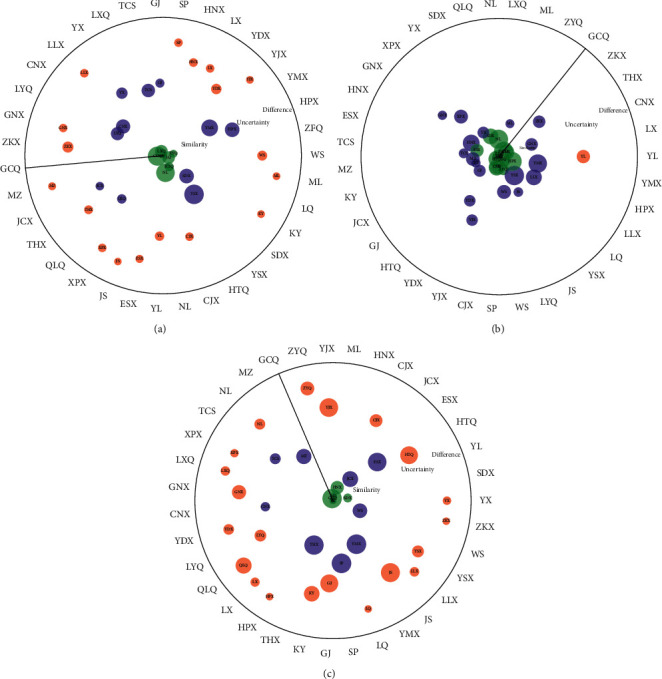
The results of climate, altitude, and chemical compositions with comparison of other locations, using GCQ as an example. (a), (b), (c) The comparative results of climate, altitude, and chemical compositions in GCQ and 34 other locations. In the plot, the value in the most central and extreme edge of the circle is 1 and 0, respectively. The closer it is to the center of the circle, the more similar it is to the characteristics of the samples in GCQ. If GCQ is closer to the center of the circle, it means that GCQ has high autocorrelation.

In the next step, we further adopted radar chart analysis to visually analyze the results of a multidimensional confidence ellipse, which helps to intuitively show the differences of data in climate, altitude, and chemical of tobacco leaves among locations. Similarly, using the sample classification of GCQ as an example, the radar chart is used to compare the differences between the samples of GCQ and all other locations in terms of climate, altitude, and chemicals, as shown in Figure 7. The details of results are introduced in the figure captions. These results well exemplify the capacity of PCA/MCEA method to transform the exploratory results of PCA into quantitative expressions of correlations between different classes of samples and further analyze the quantitative correlations of different independent variables influencing factors on dependent variables.

### 4.3. Results of Conventional Methods

**Figure 8:**
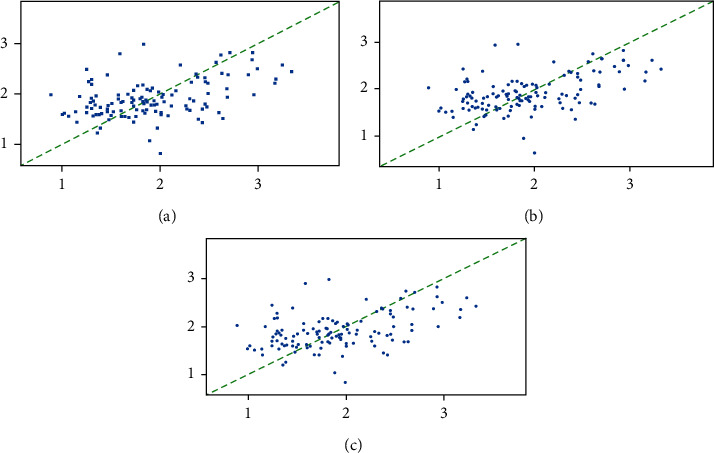
The results obtained from regression to the chemical element K. (a), (b), (c) The results of stepwise regression, PLS regression, and SVR analysis, respectively.

As introduced above, the PCA/MCEA method was used to explain the effect of climate and altitude on the chemical compositions in tobacco leaves. In this section, three traditional regression methods were attempted to be used for building of more accurate and reliable models to the same data. With the help of the leave-one-out (LOO) method for validation, stepwise regression, PLS regression, and SVR analysis were utilized to construct a quantitative model for possible accurate prediction of chemicals, which are obtained by using the model between climate, altitude, or other independent variables and the known chemical indices of tobacco leaves. Here, the influence of climate and altitude on the content of element K (potassium) of tobacco leaves was demonstrated with the results given in Figure 8. The models of climate, altitude, and content of K were constructed by the three methods with the R-squared of 0.1336, 0.1386, and 0.1431, respectively. Obviously, the performance of such models was not good enough to qualitatively discover the correlation among datasets, and thus, it has poor predictive ability for model generalization. That is to say, it is almost uninformed from the results of regression modeling. The models of the contents of total sugar, reducing sugar, nicotine, and chlorine in tobacco leaves were established with all R-squared less than 0.1. These results fully illustrate the limitations of regression methods to be used for effective finding of the relationship of independent and dependent variables, such as climate and altitude, and chemicals in tobacco leaves. The performance of modeling further shows the difficulty of conventional methods for modeling prediction of data with weak correlation, such as climate and altitude and chemical compositions of tobacco leaves. It has high challenges to find a quantitative correlation by using regression analysis, while the influence of potential factors on independent variables is too complicated with limited prior knowledge.

In this work, the PCA/MCEA method was constructed based on division and pretreatment of original data, and PCA is first performed for dimension reduction on different data obtained from samples classification. Then, the relationship of samples is constructed by using multidimensional confidence ellipse analysis through finding the samples of different classes existing in the inside and out of these ellipses. The comparative analysis of samples between different groups can be achieved for quantitative analysis of difference and similarity between independent and dependent variables. This largely helps to effectively explore the hidden information of weak correlation between datasets. The dilemma of the traditional methods for quantitative modeling is overcome for processing of data with weak correlation and low capacity for new prediction.

## 5. Conclusions

The PCA/MCEA method proposed in this work seeks to discover the weak correlation between complex data with the help of a multidimensional confidence ellipse obtained from the results of PCA operation. The common features and difference of samples between different types of samples are characterized by the number of samples existing in the overlapping or nonoverlapping areas of ellipses, which mathematically contains the characteristics of one or multiple classes of samples. The quantitative correlation between independent and dependent variables is comprehensively evaluated through individually analyzing the relationship of data pairs for information discovery. Data containing 171,516 tobacco leaves were handled as an example to deliver the strategy of the proposed method. In contrast to the conventional methods for classification and regression analysis, the results obtained from PCA/MCEA are more helpful to generate rich and informative conclusions. It can be also widely used for more types of complicated datasets with low but potential correlations.

## Figures and Tables

**Table 1:** The details of the example data of climate, altitude, and chemical compositions, respectively.

No.	Data	Name	Description
1	**X**1	Climate	The 14 climate factors include sunshine hours, temperature, altitude, precipitation, and so on
2	**X**2	Altitude	The 4 altitude factors include minimum altitude, maximum altitude, average altitude, main altitude, and so on
3	**Y**1	Chemicals	The 5 chemical indices include total sugar, reducing sugar, nicotine, potassium, and chlorine
4	**D**	Description	The 7 description information includes county, city, village committee, topography, soil type, and planting cycle year

Note: the number of objects of raw data is 171,516.

## Data Availability

The data used to support the findings of this study are available from the corresponding author upon request.
